# *Gbvdr6*, a Gene Encoding a Receptor-Like Protein of Cotton (*Gossypium barbadense*), Confers Resistance to Verticillium Wilt in *Arabidopsis* and Upland Cotton

**DOI:** 10.3389/fpls.2017.02272

**Published:** 2018-01-17

**Authors:** Yuwen Yang, Tianzi Chen, Xitie Ling, Zhengqiang Ma

**Affiliations:** ^1^The Applied Plant Genomics Laboratory of Crop Genomics and Bioinformatics Center, National Key Laboratory of Crop Genetics and Germplasm Enhancement, Nanjing Agricultural University, Nanjing, China; ^2^Provincial Key Laboratory of Agrobiology, Jiangsu Academy of Agricultural Sciences, Nanjing, China

**Keywords:** *Gbvdr6*, *Gossypium*, resistance, signaling pathway, Verticillium wilt

## Abstract

Verticillium wilt is a soil-borne disease that can cause devastating losses in cotton production. Because there is no effective chemical means to combat the disease, the only effective way to control Verticillium wilt is through genetic improvement. Therefore, the identification of additional disease-resistance genes will benefit efforts toward the genetic improvement of cotton resistance to Verticillium wilt. Based on screening of a BAC library with a partial *Ve* homologous fragment and expression analysis, a *V. dahliae*-induced gene, *Gbvdr6*, was isolated and cloned from the Verticillium wilt-resistant cotton *G. barbadense* cultivar Hai7124. The gene was located in the gene cluster containing *Gbve1* and *Gbvdr5* and adjacent to the Verticillium wilt-resistance QTL hotspot. *Gbvdr6* was induced by *Verticillium dahliae* Kleb and by the plant hormones salicylic acid (SA), methyl jasmonate (MeJA) and ethephon (ETH) but not by abscisic acid (ABA). Gbvdr6 was localized to the plasma membrane. Overexpression of *Gbvdr6* in *Arabidopsis* and cotton enhanced resistance to *V. dahliae*. Moreover, the JA/ET signaling pathway-related genes *PR3, PDF 1.2, ERF1* and the SA-related genes *PR1* and *PR2* were constitutively expressed in transgenic plants. *Gbvdr6*-overexpressing *Arabidopsis* was less sensitive than the wild-type plant to MeJA. Furthermore, the accumulation of reactive oxygen species and callose was triggered at early time points after *V. dahliae* infection. These results suggest that *Gbvdr6* confers resistance to *V. dahliae* through regulation of the JA/ET and SA signaling pathways.

## Introduction

*Gossypium hirsutum* (also known as upland or Mexican cotton) is the most widely cultivated cotton species in the world and is generally susceptible to Verticillium wilt, which is one of the most destructive diseases caused by the soil-borne fungus *Verticillium dahliae* Kleb (Zhou et al., 2013; Zhang J. et al., 2014). In contrast, *Gossypium barbadense* (also known as Sea Island, Pima, or Egyptian cotton) is usually resistant or tolerant to Verticillium wilt (Zhang J. et al., 2014). The genetic improvement of *G. hirsutum* through introgression from *G. barbadense* is an effective way to control Verticillium wilt (Zhang J. et al., 2012; Fang et al., 2013; Zhou et al., 2013). However, hybrid breakdown and sterility in interspecific crosses between *G. hirsutum* and *G. barbadense* are real obstacles to the efficient use of Verticillium wilt resistance from *G. barbadense* (Zhang J. et al., 2014). In addition to conventional breeding, the genetic engineering of disease-resistance genes is an effective means of controlling Verticillium wilt in cotton (Wang et al., 2004; Rajasekaran et al., 2005; Tohidfar et al., 2005; Miao et al., 2010; Parkhi et al., 2010; Tian et al., 2010). By screening a cotton bacterial artificial chromosome library using a cotton-expressed sequence tag that shares 48% similarity with the Verticillium wilt-resistance gene *Ve1*, we identified forty independent positive clones and isolated two Verticillium wilt-resistance genes encoding receptor-like proteins (RLPs) (Zhang B. et al., 2012; Yang et al., 2015a). Other proteins, including mitogen-activated protein kinases, WRKY transcription factors, MYB transcription factors, major latex proteins, polyamine oxidases, receptor-like kinase, BRI1-associated receptor kinase, and subtilase are also involved in the defense response of cotton to *V. dahliae* (Gao et al., 2013; Li et al., 2014; Zhang X. et al., 2014; Jun et al., 2015; Mo et al., 2015; Yang et al., 2015a; Cheng et al., 2016; Duan et al., 2016). However, the control of Verticillium wilt is extremely difficult due to the persistence of the microsclerotia in soil and the lack of an effective chemical means of combating the pathogens (Klosterman et al., 2009). Therefore, the identification of additional disease-resistance genes will benefit efforts in the genetic improvement of cotton's resistance to Verticillium wilt.

RLPs, two examples of which are the tomato *Cladosporium fulvum* (*Cf*) resistance genes and the tomato Verticillium wilt-resistance Ve locus (Kawchuk et al., 2001; Kruijt et al., 2005), typically possess an extracellular leucine-rich repeat (eLRR), a single transmembrane domain, and a short cytoplasmic C terminus. The Ve locus comprises two closely linked inversely oriented genes, Ve1 and Ve2, that share 84% amino acid identity. Whereas Ve1 mediates Verticillium resistance by recognizing the *V. dahliae* Ave1 effector, Ve2 has no resistance function in tomato or transgenic *Arabidopsis* (Fradin et al., 2009, 2011; Jonge et al., 2012). Domain swaps between Ve1 and Ve2 showed that the first 30 eLRRs of Ve1 can be replaced by those of Ve2, whereas the eLRR30-eLRR35 region and the C-terminus of Ve1 are crucial for Verticillium wilt resistance and cannot be replaced by those of Ve2 (Fradin et al., 2014). Mutational analysis of Ve1 further revealed that the C1 domain eLRR1-eLRR8 and eLRR20-eLRR23, the C2 domain, and the C3 domain eLRR32-eLRR37 are required for Ve1 functionality, whereas the GxxxG motif in the transmembrane domain and two putative endocytosis motifs in the C-terminus are not (Zhang Z. et al., 2014). The chaperones HSP70 binding proteins (BiPs) and a lectin-type calreticulin (CRT) were verified to be involved in *Ve1*-mediated resistance to Verticillium (Liebrand et al., 2014).

In response to infection by pathogens, plants have evolved a series of inducible defenses, including the induction of the hypersensitive response (HR), the formation of reactive oxygen species (ROS), the deposition of callose, and the production and accumulation of antimicrobial proteins, phytoalexins, and PR proteins (Luo et al., 2014). *Ve1*-mediated Verticillium wilt resistance triggers an HR in tomato (*Solanum lycopersicum*) and *Nicotiana tabacum* (Jonge et al., 2012; Zhang et al., 2013a), generates hydrogen peroxide (H_2_O_2_) and increases the activities of peroxidase, phenylalanine ammonia lyase, and lignins (Gayoso et al., 2010). The overexpression of other RLPs and polyamine oxidase genes in transgenic *Arabidopsis* plants has also been shown to increase the levels of callose, H_2_O_2_, salicylic acid and phytoalexin during *V. dahliae* infection (Zhang B. et al., 2012; Mo et al., 2015; Yang et al., 2015a).

Genes with homology to *Ve* have been identified and cloned in our laboratory using screening of BAC clones combined with the genome walking method (Zhang B. et al., 2012; Yang et al., 2015b). A partial fragment of a *Ve*-homologous gene was amplified from the genomic DNA of H7124 using primers designed according to a cotton EST (TC121084) in the gene index. The amplified sequence was subsequently used as a probe to screen a *G. hirsutum* cv. Maxxa BAC library, and 40 positive clones were identified (Tomkins et al., 2001). The expression patterns of these genes after inoculation of the plants with *V. dahliae* were analyzed by qRT-PCR, and *Gbvdr6* was found to be activated. Therefore, *Gbvdr6* was considered to confer resistance to *V. dahliae* and was chosen for further analysis. *Gbvdr6*-overexpressing *Arabidopsis* and cotton showed enhanced resistance to *V. dahliae*. Interestingly, the transgenic plants were less sensitive than wild-type plants to MeJA, while at the same time, the JA/ET signaling pathway was induced. H_2_O_2_ production and callose deposition were also found to be enhanced in *Gbvdr6* transgenic plants at the early infection stage.

## Materials and methods

### Plants, *V. dahliae* strain and inoculation method

Seedlings of the *G. barbadense* cultivar Hai7124, which is highly resistant to *V. dahliae* (Yang et al., 2008), were grown in chambers under greenhouse conditions. The *Arabidopsis thaliana* ecotype Columbia-0 was cultured in pots under controlled conditions (temperatures of 25°C during the day and 20°C at night, 60–70% relative humidity, and light intensity of 200 μmol/m^−2^/s^−1^ in a 16/8 h photoperiod). A non-defoliating isolate of *V. dahliae*, BP2 (Yang et al., 2008), was activated on potato dextrose agar and cultured in liquid Czapek medium at 25°C. Before inoculation, the spore concentration of *V. dahliae* was determined by counting its spores under a microscope. For *V. dahliae* inoculation, seedlings of Hai7124 at the 2-leaf stage were inoculated by root irrigation with 10 mL of liquid containing 1 × 10^7^ spores per pot (Zhang B. et al., 2012); plantlets of *Arabidopsis thaliana* were uprooted and dipped for 1 min in a suspension containing 1 × 10^7^ spores/mL and replanted in vermiculite (Fradin et al., 2011). For hormone treatment, the roots of cotton seedlings at the 4-leaf stage were immersed in 1 mM salicylic acid (SA), 5 mM ethephon (ETH), 100 μM abscisic acid (ABA) or 100 μM methyl jasmonate (MeJA), (Chen et al., 2014; Camacho-Cristóbal et al., 2015). Stem tissues from *V. dahliae*-infected plants and root tissues from hormone-treated plants were harvested at appropriate times for RNA extraction.

### Isolation of *Gbvdr6*

The gene cloning method used in this study followed our previously described protocol (Zhang B. et al., 2012; Yang et al., 2015a). Briefly, forty BAC clones were obtained by screening a *G. hirsutum* cv. Maxxa BAC library against a cotton EST (TC121084) that is highly homologous to the tomato *Ve1* gene (Zhang B. et al., 2012). Partial sequences of the *Ve1*-homologous gene in one BAC clone were amplified using the conserved primers VeF1008 (5′-ttgagcaattgacaagaatagagct-3′) and VeR2731 (5′-tgaccctgtgaaagcattatgtga-3′), and the full sequence of this BAC clone was obtained by genome walking using the specific primers 5′-ttagtgtaagtaagactgagggaag-3′ and 5′-gcaactcggtcagtggcaataaa-3′ and 5′-ttcccaatgtccgtgtttgaact-3′ and 5′-ggcaaatcccaattcttaaccca-3′ for 5′ and 3′ sequence amplification, respectively. To clone its ortholog in *G. barbadense* cv. Hai7124, the specific primers F-*Xba*I (5′-tgatctagactcaacctagtgccattgttatc-3′) and R-*Sma*I (5′-tgacccgggaatcccaatagttgctaggtcct-3′), which introduce a 5′ *Xba*I site and a 3′ *Sma*I site, respectively, were designed according to the full sequences of the *Ve1*-homologous gene in *G. hirsutum*. Polymerase chain reaction (PCR) was conducted with Hai7124 cDNA and DNA as templates, respectively. The PCR program consisted of 3 min at 94°C followed by 32 cycles of denaturation for 45 s at 94°C, annealing for 45 s at 56°C, and extension for 3 min at 72°C. The PCR product was cloned into the pGEM-T vector (Promega) and sequenced after agarose gel electrophoresis and purification using a QIAquick PCR Purification Kit (Qiagen).

Alignments of nucleotide and amino acid sequences were conducted using BioEdit software. A GenBank BLASTX search was performed on the website of the National Center for Biotechnology Information (http://www.ncbi.nlm.nih.gov). Phylogenetic analysis was performed using MEGA6 software (Tamura et al., 2013). The putative motif and domains were analyzed with SignalP 4.1 (http://www.cbs.dtu.dk/services/SignalP/), InterProScan (http://www.ebi.ac.uk/interpro/scan.html), and SMART (http://smart.embl-heidelberg.de/). The sequences of the SSR markers that flank Verticillium wilt-resistance quantitative trait loci (QTL) in cotton were downloaded from the CottonGen (https://www.cottongen.org) and CottonQTLdb (http://www2.cottonqtldb.org:8081/search) websites. The physical locations of genes and markers were determined by anchoring their sequences onto the island cotton upland cotton (*G. hirsutum*) cv. TM-1 genome (Zhang et al., 2015) through BLASTN with an *E*-value = 1.0E-100 for genes and an *E*-value = 1.0 for markers. To confirm the physical positions of the marker primers, the distance between one forward primer hit and one reverse primer hit was determined to be within 100–500 bp in the same chromosome.

### Expression pattern analysis of *Gbvdr6*

To characterize the expression patterns of the *Gbvdr6* gene in various tissues in response to *V. dahliae* infection and phytohormones, total RNA was extracted from root, stem and leaf tissues using an RNAiso Kit (TaKaRa) and transcribed into cDNA using a Primscript RT-PCR kit (TaKaRa) according to the manufacturer's instructions. A quantitative reverse transcription-polymerase chain reaction (qRT-PCR) was performed using a SYBR Premix ExTaqTM II Kit (TaKaRa) in a real-time PCR thermal cycler (qTOWER 2.0/2.2, Analytik Jena, Germany). The *Gbvdr6*-specific primers for qRT-PCR were F2952 (5′-tcgtcaccacctaaagaagacag-3′) and R3097 (5′-cacgatcgacacgctcaaaatac-3′), which corresponded to the 3′ end region of *Gbvdr6* mRNA. The cotton polyubiquitin 14 gene served as an internal control, with the specific primers 5′-caacgctccatcttgtcctt-3′ and 5′-tgatcgtctttcccgtaagc-3′ (Artico et al., 2010). The PCR program consisted of an initial denaturation step of 1 min at 95°C followed by denaturation for 15 s at 95°C, annealing for 20 s at 60°C, and extension for 20 s at 72°C for 40 cycles. All qRT-PCR results are expressed as the relative expression levels determined using three biological replicates. The *Gbvdr6* promoter cassette was constructed by inserting the 1.66-Kb fragment upstream of the start codon of Hai7124 into PbI101 using the primers pGbvdr6HinIIIF (5′-tccaagcttcagacttaccaggagataacattc-3′) and pGbvdr6BamHIR (5′-agtggatccaatacaacaaagaattatgaagaa-3′). Histochemical localization of GUS activity in *Arabidopsis* transfected with the *Gbvdr6* promoter construct was performed as previously described (Jefferson et al., 1987). The relative expression levels were calculated using the 2^−ΔΔCT^ method (Livak and Schmittgen, 2001). The relative transcript levels of *Gbvdr6* were normalized to the transcript levels of the polyubiquitin 14 (UBQ14) gene. In each case, three technical replicates were performed for each of at least three independent biological replicates.

### Subcellular localization analysis of Gbvdr6

*Gbvdr6* was PCR-amplified using the primers 5′-cggggtaccatgaggatttcactcttttc-3′ and 5′-cgcggatccggtcctcctttggttctg-3′, which introduce a 5′ *Kpn*I site and a 3′ *Bam*HI site, respectively, and fused to the N-terminus of GFP in the pBinGFP4 vector under the control of the CaMV35S promoter (Liu et al., 2014). The Gbvdr6-GFP fusion and the plasma membrane marker mCherry (Nelson et al., 2007) were co-agroinfiltrated into *N. benthamiana* leaves. Fluorescence was imaged at 48 h post-infiltration using a Zeiss LSM710 confocal microscope (Zeiss Microsystems) at specific excitation and emission wavelengths (GFP, 488 and 495–530 nm; mCherry, 587 and 600–650 nm).

### Pathogen inoculation assay of transgenic *Arabidopsis* and cotton

A binary vector containing an overexpression cassette of *Gbvdr6* under control of the CaMV35S promoter was transformed into *Arabidopsis* Columbia and *Gossypium hirsutum* var. 03298 by floral dip and *Agrobacterium*-mediated transformation of cotton hypocotyl, respectively (Umbeck et al., 1987; Bent, 2006). The expression of *Gbvdr6* in transgenic plants was investigated by qRT-PCR using the specific primers F1785 (5′-gcaacaagtctcgagtacctaaat-3′) and R2140 (5′-ccaagagacacgttcatctgaaag-3′). The *Arabidopsis* β-tubulin gene and the cotton polyubiquitin 14 gene were used as internal controls (Hiratsu et al., 2003).

The Verticillium wilt resistance of T3 transgenic *Gbvdr6 Arabidopsis* was evaluated based on the phenotypes of *V. dahliae*-inoculated plants using 3 disease grades: healthy, stunted or dead. The T3 plants of transgenic cotton were challenged by *V. dahliae* inoculation according to a previously described method (Zhang B. et al., 2012). The *V. dahliae*-inoculated cotton plants were scored and classified into five grades as described in our previous report (Zhang B. et al., 2012). The quantification of *V. dahliae* biomass was performed as described previously (Fradin et al., 2011).

### Expression analysis of defense-related genes in transgenic *Arabidopsis*

The expression levels of seven pathogenesis-related genes in *Arabidopsis* (*PR1, PR2, PR3, PR5, PDF1.2, ERF1*, and *GST2*) (Mazarei et al., 2007) were investigated 7 days after *V. dahliae* inoculation by qRT-PCR as described above with the β-tubulin gene as a reference (Hiratsu et al., 2003). The primers used for qRT-PCR were as follows: PR1-F (5′-gctcttgtaggtgctcttgttcttccct-3′) and PR1-R (5′-ctggttgtgaacccttagataatcttgtgg-3′); PR2-F (5′-caatctcccttgctcgtgaatctctaccc-3′) and PR2-R (5′-cgttatcaacagtggactgggcgg-3′); PR3-F (5′-ttaacggcctcctcgaagctgctattt-3′) and PR3-R (5′-cgcaacataaacagtgaaacatcattggaa-3′); PR5-F (5′-caagaacgcttgccctgacgccta-3′) and PR5-R (5′-gctccggtacaagtgaaggtgctcgtt-3′); PDF1.2-F (5′-caagtgggacatggtcaggggtt-3′) and PDF1.2-R (5′-cacttgtgtgctgggaagacatagttgc-3′); ERF1-F (5′-agcagtccacgcaacaaacctat-3′) and ERF1-R (5′-aaagcgactcttgaactctctcc-3′); GST2-F (5′-ccagcttccgagaaggttcagtgagaa-3′) and GST2-R (5′-gaaattgggcaatgagaaagccgctt-3′); and β-tubulin-F (5′-cgtggatcacagcaatacagagcc-3′) and β-tubulin-R (5′-cctcctgcacttccacttcgtcttc-3′).

### Detection of reactive oxygen and callose formation in transgenic cotton

Cotton roots were inoculated with a solution of *V. dahliae* (1 × 10^7^ conidia/ml) and incubated at 25°C in a humidified incubator for 5 days. Histochemical assays of H_2_O_2_ accumulation and callose deposition in infected roots were performed according to the method of Choi et al. (2012); the results were visualized with a fluorescence microscope under bright light and UV light, respectively.

### Tolerance of *Arabidopsis* transgenic plants to MeJA

The seeds of WT and *Gbvdr6*-overexpressing lines were surface-sterilized with 70% ethanol for 30 s followed by exposure to 5.6% NaClO for 5 min; the seeds were then washed at least five times with sterile distilled water. The seeds were placed in 1/2 MS medium with or without 20 μM MeJA. Germination rates were calculated as the percentage of seeds with radicles protruding through the seed coat. The assays were replicated three times with 50 seeds each time. The root lengths of the seedlings (the distance to the root tip) were also measured using a ruler; at least 50 seeds were measured in each of three replicates (Gibson and Todd, 2015).

### Physiological analysis of Gbvdr6-overexpressing plants

Approximately 1 g of fresh root tissue from *V. dahliae*-inoculated or control plants was ground thoroughly in liquid nitrogen. The supernatant was used for analysis of PAL (EC 4.3.1.5) and CAT (EC 1.11.1.6) activity according to the instructions provided with the kit (Nanjing Jiancheng Bioengineering Institute). All enzyme activities are expressed as units mg^−1^ protein.

### Statistical analysis

All statistical analyses were performed using the “ANOVA analysis” software designed by the Nanjing Agricultural University. The Chi-square test and Fisher's exact test were used to evaluate the Verticillium wilt resistance of transgenic *Gbvdr6 Arabidopsis* and cotton, respectively. The Kruskal-Wallis test was conducted using the R language to analyze the expression of *Gbvdr6* by the induction of *V. dahliae*, and Duncan's multiple range test was performed on the basis of the ANOVA analysis in SPSS 19.0 to compare the expression of *Gbvdr6* in different organs and the expression of pathogenesis-related genes in transgenic *Arabidopsis*. In the graphs, asterisks and different letters indicate significant differences between treatments (^*^P < 0.05; ^**^P < 0.01), and letters shared in common between or among the experimental groups indicate no significant difference.

## Results

### *Gbvdr6* is located in a gene cluster adjacent to the verticillium wilt-resistance QTL hotspot

**Figure 1:**
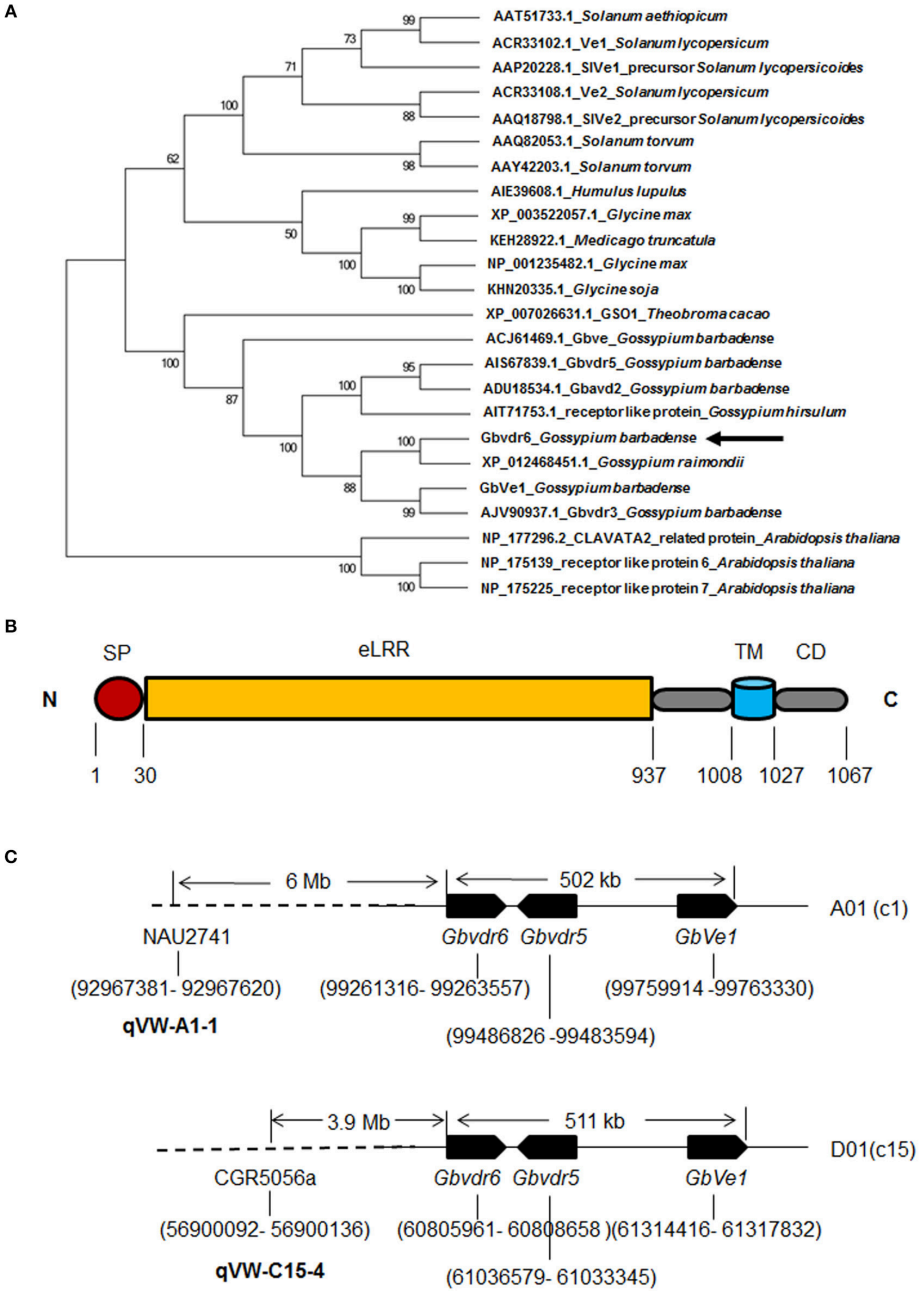
Phylogenetic and structural analysis of *Gbvdr6* gene. **(A)** Phylogenetic relationship of Gbvdr6 with other Ve-like proteins by MEGA6 software with the neighbor-joining (NJ) algorithm under 1,000 replicates of bootstrap. The numbers on the internal nodes are the percentage bootstrap support values. **(B)** Schematic diagram of *Gbvdr6* protein domain architecture showing signal peptide (SP) at N-terminus, followed by extracellular leucine-rich repeat (eLRRs), transmembrane (TM) domain and cytoplasmic domain (CD) at C-terminus. The numbers indicate the domain regions. **(C)** Schematic diagram of physical locations of *Gbvdr6, Gbvdr5*, and *GbVe1* and the SSR markers flanking the known Verticillium wilt-resistant QTLs in the chromosomes of tetraploid cotton. The numbers in brackets indicate the physical positions in the chromosome. NAU2741 and CGR5056a are the SSR markers that flank Verticillium wilt resistance QTLs *qVW-A1-1* and qVW-C15-4 in the A01(c1) and D01(c15) chromosomes, respectively.

*Gbvdr6* cDNA consists of 3,328 bp (GenBank accession: KT809405), including an ORF of 3,204 bp that encodes a polypeptide comprising 1,067 amino acid (aa) residues with a calculated molecular weight of 119.28 kDa and a predicted isoelectric point of 6.97. The 3,328 bp of cDNA sequence were mapped into the genomic region of 41706732-41710044 of the At_chr9 chromosome of upland cotton (*G. hirsutum*) cv. TM-1 (Li et al., 2015) with 99% identity and a 3-bp gap (Figure S1), indicating that no intron exists in this gene. The sequences obtained by PCR amplification of the Hai7124 DNA template further confirmed that *Gbvdr6* contains no intron. A BLASTX search of the non-redundant protein sequences database of NCBI revealed that the *Gbvdr6* cDNA shared the highest identity (93%) with a receptor-like protein in *G. raimondii* and with a series of receptor-like proteins associated with Verticillium wilt disease resistance in *G. barbadense, Medicago truncatula, Humulus lupulus, Glycine max, Solanum torvum*, and *Solanum lycopersicoides*. Phylogenetic analysis further demonstrated that Gbvdr6 is most closely related evolutionarily to cotton Verticillium wilt disease-resistance proteins (Figure 1A). SMART analysis indicated that Gbvdr6 possesses distinct domains corresponding to those of receptor-like proteins, including a signal peptide, multiple LRRs, a transmembrane domain and a cytoplasmic region (Figure 1B and Figure S1).

Sequence mapping through a BLASTN search of the genome of upland cotton (*G. hirsutum*) cv. TM-1 (Zhang et al., 2015) revealed that *Gbvdr6* had two homologs in *G. hirsutum*. One was on the A01 (C1) chromosome, and the other was on the D01 (c15) chromosome. Similarly, *GbVe1* and *Gbvdr5* had homologs on the A01 (C1) and D01 (c15) chromosomes of *G. hirsutum*. The physical positions of these cotton RLPs further revealed that the homologs of *Gbvdr6* were physically clustered with those of *GbVe1* and *Gbvdr5* on the A01 or D01 chromosome of upland cotton (Figure 1C). At the same time, one Verticillium wilt-resistance QTL hotspot, C15-VW-QTL-rich-1, including qVW-C15-2, qVW-C15-3, and qVW-C15-4, and four QTLs including qVW-A1-1, qRV07DF2-A1-1, qDL52T2-c15, and qRD8092-D1-1 were detected on the A01 (C1) and D01 (c15) chromosomes of tetraploid cotton, respectively (Ning et al., 2013; Fang et al., 2014; Wang et al., 2014; Shi et al., 2016). The flanked markers of these QTLs were anchored to the TM-1 genome through BLASTN. The physical positions of these markers on the chromosome further revealed that the SSR marker CGR5056a, which was linked to qVW-C15-4, was approximately 3.9 Mb from the gene cluster of *Gbvdr6*-Gbvdr5-*GbVe1* homologs on the D01 (C15) chromosome, whereas the SSR marker NAU2741 that flanked qVW-A1-1 was only 6 Mb upstream of the gene cluster on the A01 (C1) chromosome (Figure 1C).

### *Gbvdr6* is highly expressed in root and is induced by *V. dahliae*, SA, MeJA, and ETH treatment

**Figure 2:**
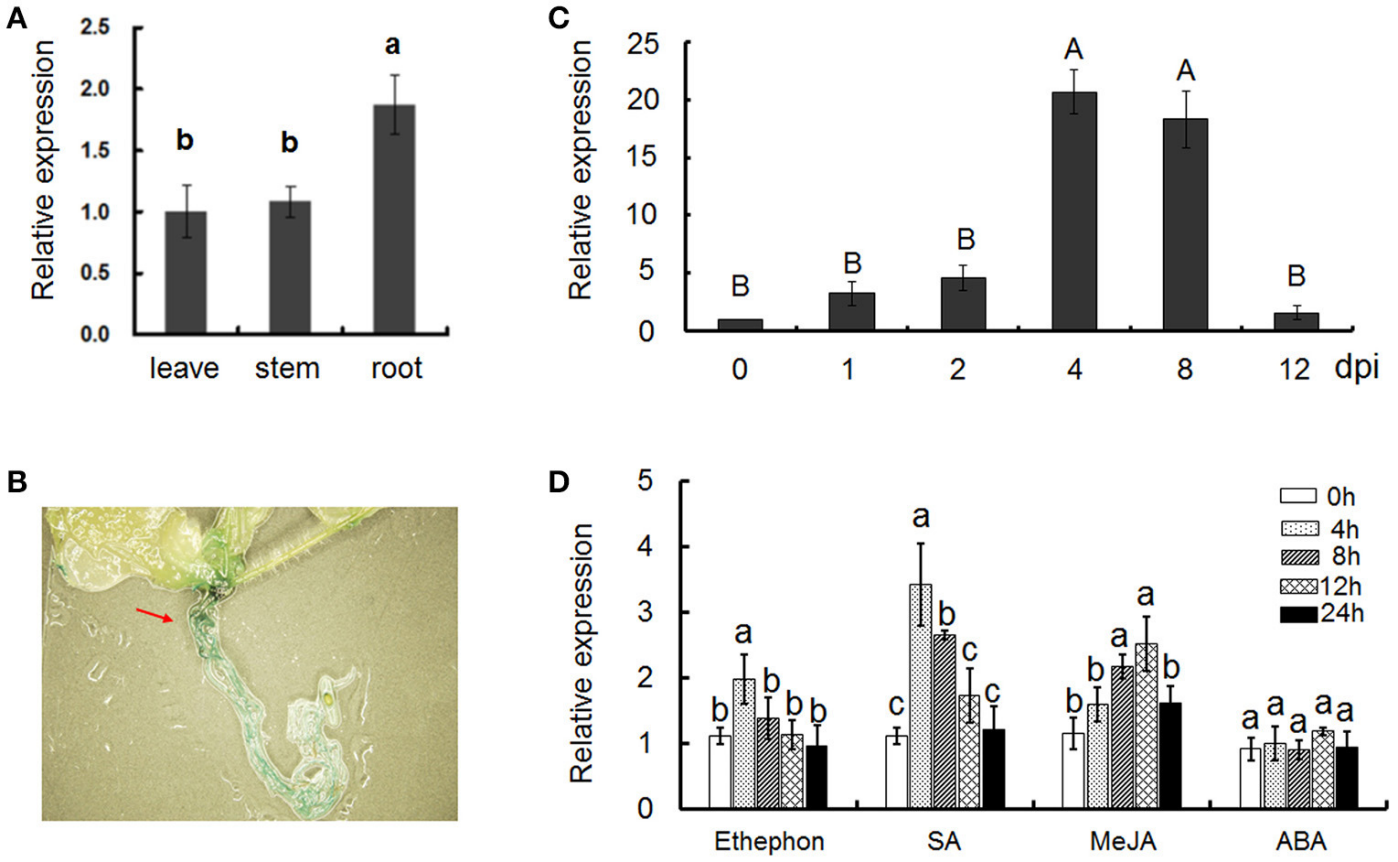
Expression pattern analysis of the *Gbvdr6* gene. **(A)** The transcript levels of *Gbvdr6* in different tissues of Hai7124. Values were expressed as fold changes of transcript levels in the different tissues with respect to that of leaves with the 2^−ΔΔCT^ Method. Error bars represented SE of three biological replicates. Duncan's multiple range test was conducted, and the different letters in graphs indicate significant differences between treatments (*P* < 0.05). **(B)** Gus activities in transgenic *Arabidopsis* plantlets containing the pGbvdr6: Gus construct. **(C)** The expression patterns of *Gbvdr6* in response to infection by *V. dahliae*. The transcript levels of *Gbvdr6* were measured by real-time reverse-transcription PCR with the *UBQ14* gene as the internal control. Values were expressed as fold changes of transcript levels in the *V. dahliae* inoculated samples at fixed point of time with respect to that in non-inoculated samples at 0 dpi with the 2^−ΔΔCT^ Method. Error bars represented SE of three biological replicates. Kruskal-Wallis test was conducted, and the different letters in graphs indicate significant differences between treatments (*P* < 0.01). **(D)** The expression patterns of *Gbvdr6* in response to phytohormones. Values were expressed as fold changes of transcript levels in the phytohormones treated root samples at fixed point of time with respect to the transcript levels in the mock samples. Error bars represented SE of three biological replicates and the different letters in graphs indicate significant differences after treatments (*P* < 0.05).

The expression patterns of *Gbvdr6* in various cotton tissues were examined by quantitative real-time PCR (qRT-PCR) analysis. *Gbvdr6* was ubiquitously expressed in all tested tissues, and its transcripts accumulated to the highest level in root (Figure 2A). *Gbvdr6* promoter-driving GUS activity was also found mostly in the root of transgenic *Arabidopsis* (Figure 2B), consistent with the results of qRT-PCR. To further investigate the involvement of *Gbvdr6* in the response to *V. dahliae* infection, gene expression was analyzed after inoculation of cotton seedlings with *V. dahliae*. The expression of *Gbvdr6* was induced until 4 days post-inoculation (dpi), reached maximum levels at 4–8 dpi, and then returned to the control levels at 12 dpi (Figure 2C). SA, MeJA, and ETH but not ABA markedly increased *Gbvdr6* transcript levels in roots and the transcript levels increased during the early stages of treatment and decreased thereafter (Figure 2D).

### Gbvdr6 protein is localized on the plasma membrane

**Figure 3:**
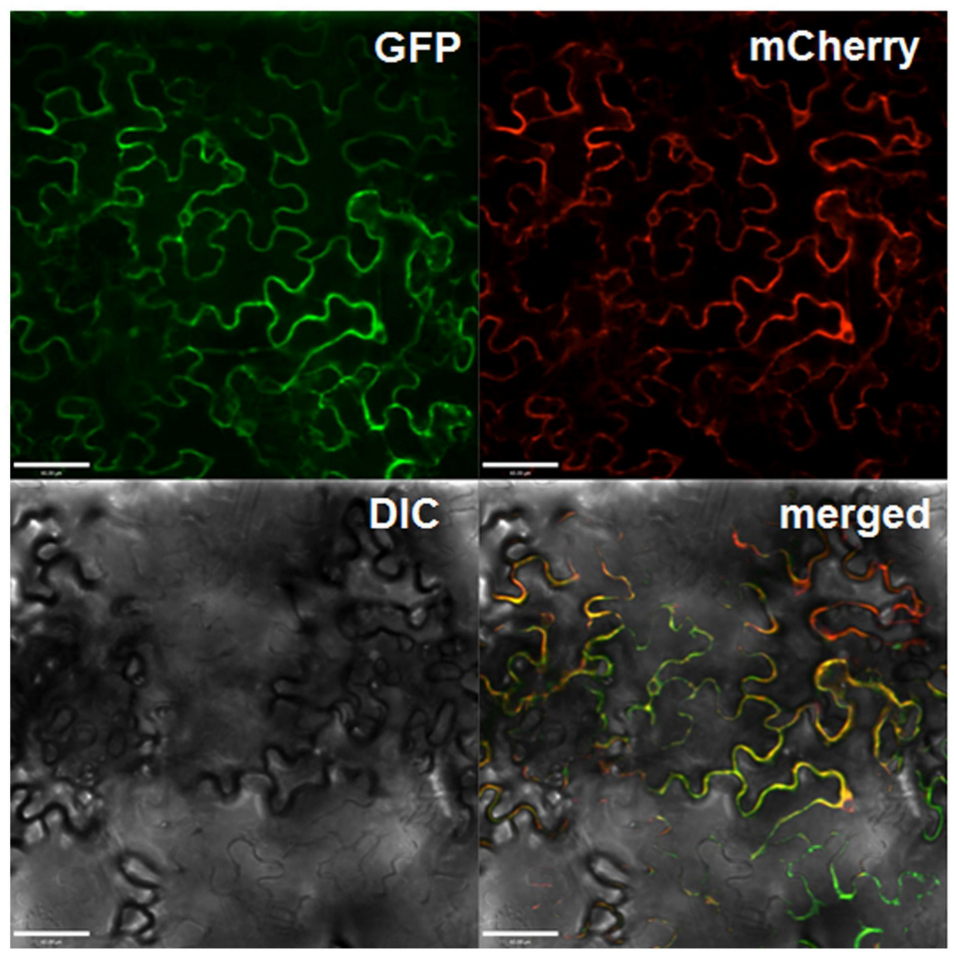
Subcellular localization of Gbvdr6 in epidermal cells of *N. tabacum* leaves. The Gbvdr6-GFP fusion was transiently co-expressed with the plasma membrane marker mCherry. The images were taken under a confocal microscope at 48 h after agro-infiltration. GFP: fluorescence of Gbvdr6-GFP fusion, mCherry: fluorescence of the plasma membrane marker mCherry, DIC, differential interference contrast; merged, a merged image. Scale bar = 60 μm.

To investigate the subcellular localization of Gbvdr6, Gbvdr6 was fused with to the N-terminus of GFP and transiently co-expressed with the plasma membrane marker mCherry in *N. benthamiana* leaves. Confocal microscopy revealed that the green fluorescent signal from the Gbvdr6-GFP fusion and the red fluorescent signal from mCherry were co-localized (Figure 3), indicating that Gbvdr6 is localized on the plasma membrane.

### Overexpression of *Gbvdr6* enhances resistance to *Verticillium* in *Arabidopsis*

**Figure 4:**
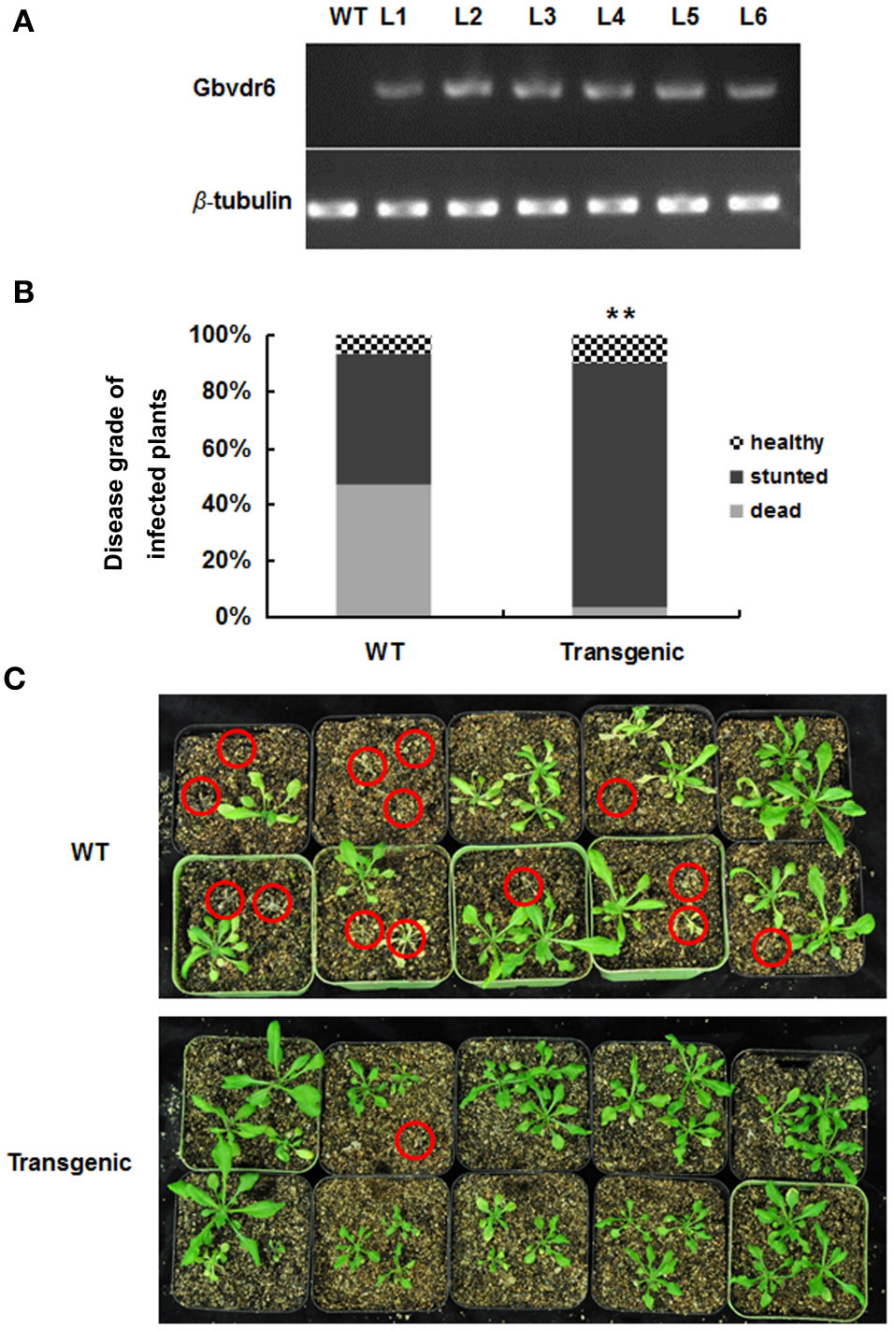
Gbvdr6 over-expressed *Arabidopsis* enhanced resistance to *V. dahliae*. **(A)** The semi-quantitative RT-PCR of *Gbvdr6* over-expressed *Arabidopsis*. The β-tubulin was the internal control, and L1-L6 are the transgenic *Arabidopsis* lines. **(B)** The numbers of healthy, stunted and dead *Arabidopsis* plants were scored and statistically analyzed. Thirty plantlets were tested in each line. The experiment was conducted twice with similar results. The chi-squared test is used to determine whether there is a significant difference. The asterisk indicated above the columns means ^**^P < 0.01. **(C)** The phenotype of the transgenic and non-transgenic cotton induced by *Verticillium dahliae*. The photos were taken at 28 days post-inoculation with *V. dahliae*. The red circles indicated the dead plants.

To explore the function of *Gbvdr6*, an overexpression cassette in the background of the binary vector pCAMBIA2301 under the control of the CaMV35S promoter was introduced into the *Arabidopsis thaliana* genome. The transgenic plants were screened on kanamycin plates and confirmed by semi-quantitative RT-PCR (Figure 4A). The T3 transgenic *Arabidopsis* lines were challenged with *V. dahliae*. At 28 dpi, the healthy, stunted and dead phenotypes of *Arabidopsis* plants in response to *V. dahliae* infection were scored. Overexpression of *Gbvdr6* in transgenic *Arabidopsis* significantly improved resistance to *V. dahliae*, the death rate of 46.7% observed in wild-type *Arabidopsis* plantlets was reduced to 3.3% in the transgenic plants (Figure 4B). However, transgenic *Gbvdr6 Arabidopsis* was not immune to *V. dahliae*, and its growth was stunted (Figure 4C).

### Overexpression of *Gbvdr6* significantly activates defense genes in *Arabidopsis*

**Figure 5:**
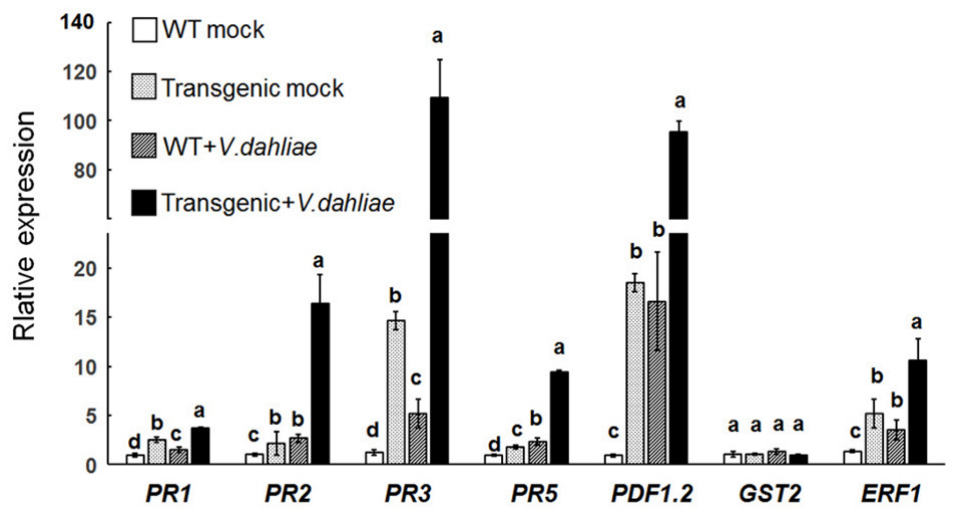
Upregulation of pathogenesis-related genes in the *Gbvdr6* over-expressed *Arabidopsis*. The expression levels of marker genes *PR1, PR2*, and *PR5* in SA signaling, *PR3, PDF1.2*, and *ERF1* in ET/JA signaling, and *GST2* in ET signaling were measured by real-time reverse-transcription PCR with β-tubulin gene as the internal control. Data were the means ± SE of three biological replicates. Duncan's multiple range test was carried out within genes, and different letters in the graphs indicate significant differences between treatments (*P* < 0.05).

The expression levels of *PR1, PR2*, and *PR5*, which are marker genes for SA signaling, those of *PDF1.2* and *ERF1*, genes involved in ET- and JA-signaling and that of *GST2*, a gene involved in ET signaling, were investigated by qRT-PCR. After mock inoculation, the selected pathogenesis-related genes *PR1, PR2, PR5, PR3, ERF1*, and *PDF1.2* were significantly upregulated in *Gbvdr6*-overexpressing *Arabidopsis* (Figure 5). The expression levels of *PR1, PR2, PR5, PR3, ERF1*, and *PDF1.2* were further elevated in plants after *V. dahliae* infection. In contrast, the ET signaling marker gene *GST2* was not affected by overexpression of *Gbvdr6* or by infection with *V. dahliae*. The transcript abundances of *PR1, PR2, PR3*, and *PR5* in the *V. dahliae*-infected transgenic plants were approximately 2.5-, 6-, 18- and 4.5-fold, respectively, those of the infected control plants. The transcript abundances of *ERF1* and *PDF1.2* increased approximately 2- and 5-fold, respectively, in the *V. dahliae*-infected transgenic plants compared to the infected control plants. These results indicate that overexpression of *Gbvdr6* significantly activates the expression of defense genes and suggests that it might be involved in the SA and JA/ET-mediated signaling pathways.

### *Gbvdr6*-overexpressing plants are insensitive to MeJA

**Figure 6:**
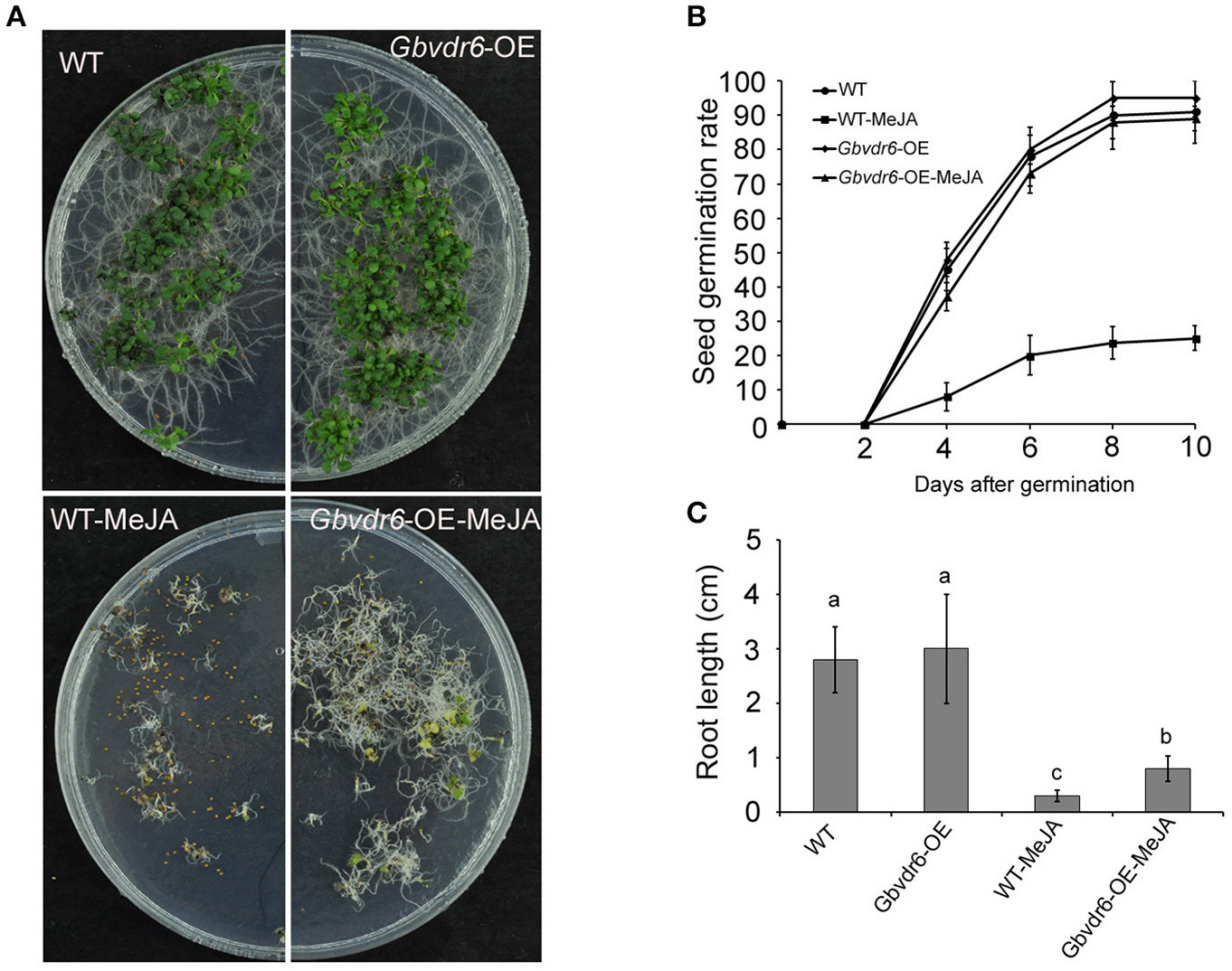
*Gbvdr6* over-expressed *Arabidopsis* is more insensitive to MeJA compared with the wild type. **(A)** The phenotype of *Gbvdr6* over-expressed *Arabidopsis* (*Gbvdr6*-OE) and wild type after MeJA treatment. The WT and *Gbvdr*6-OE are the seedlings grown on the plate without MeJA, and the WT-MeJA and *Gbvdr6*-OE-MeJA indicates the seedling on the plate with 20 um MeJA. The photos were taken at 30 days after sowing. **(B)** Assay of seed germination rate of *Gbvdr6* over-expressed *Arabidopsis* (*Gbvdr6*-OE) and wild type in the presence of exogenous MeJA. Germination rates of the seeds were analyzed at the indicated time points. The data represent means ± SD of three independent replicates with at least 50 seeds counted per replicate. **(C)** Assay of root length of *Gbvdr6* over-expressed *Arabidopsis* (*Gbvdr6*-OE) and wild type in the presence of exogenous MeJA at 30 days after sowing. Significant difference between different lines is indicated by different letters (*P* < 0.05).

Because JA is involved in regulating the response to abiotic and biotic stresses as well as in many aspects of plant growth and development, *Gbvdr6*-overexpressing and wild-type *Arabidopsis* seedlings were treated with MeJA. Although most seeds of WT plants were unable to germinate after this treatment, all of the seeds from transgenic plants germinated (Figure 6A). The seed germination rate of the WT plants fell sharply to less than 30%, whereas that of the *Gbvdr6*-overexpressing plants remained unchanged (Figure 6B). The roots of *Gbvdr6*-overexpressing plants were also significantly longer than those of WT plants after MeJA treatment (Figure 6C). This suggests that the *Gbvdr6*-overexpressing plants were less sensitive to MeJA-mediated inhibition of root growth than the control plants.

### Overexpression of *Gbvdr6* enhances *Verticillium* resistance in cotton and enhances the immune response

**Figure 7:**
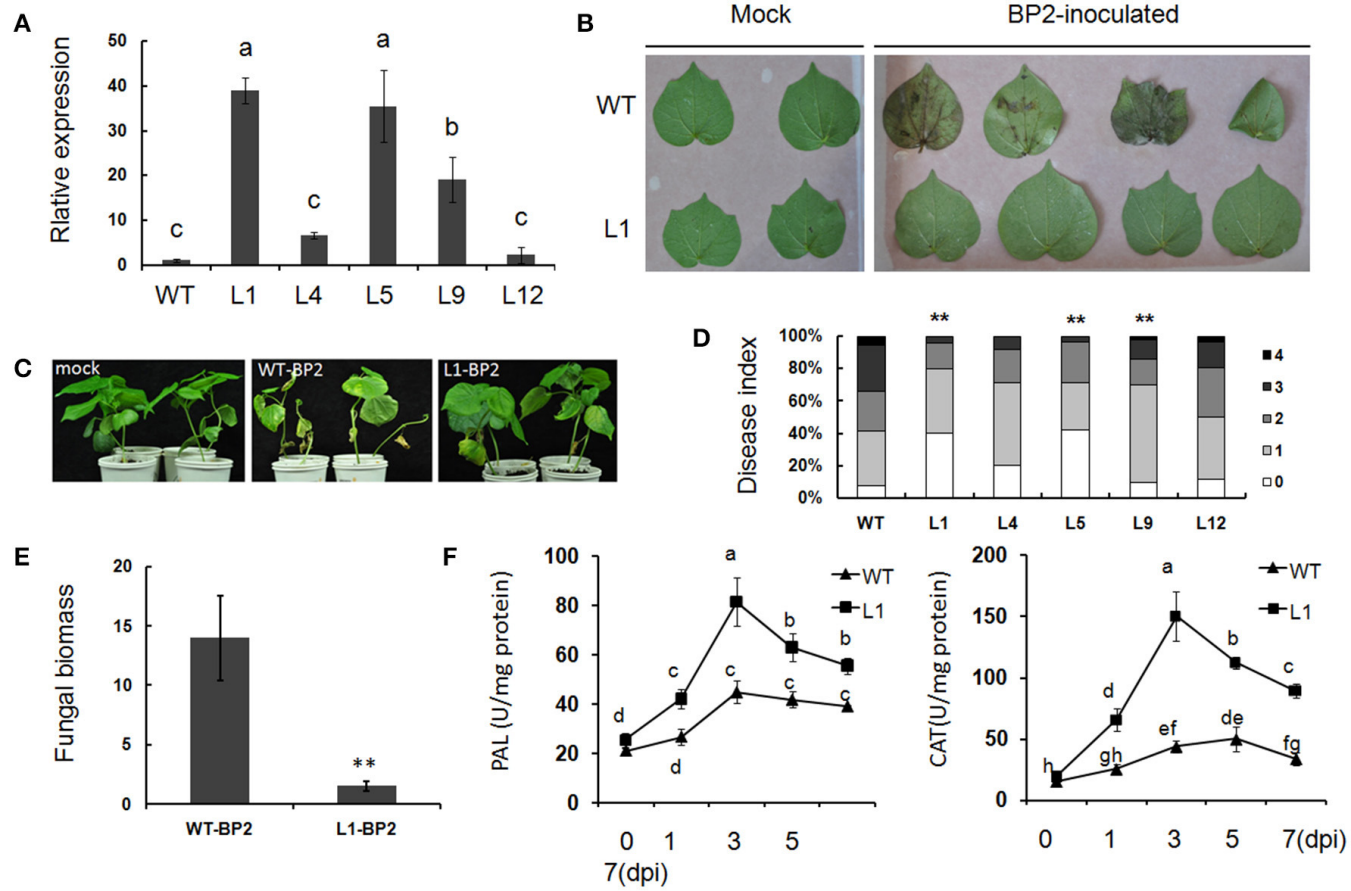
*Gbvdr6* over-expressed cotton enhanced resistance to *V. dahliae*. **(A)** Varied expressional levels of *Gbvdr6* in the transformed plant*s. Gbvdr6* relative expressional levels of the T3 generation were measured by qRT-PCR and calculated in relation to the wild type plants according to the ΔΔCt method with the *UBQ14* gene as the internal control. L1, L4, L5, L9, and L12 are the transgenic cotton lines. Different letters on the bars designate statistically significant differences (*P* < 0.05) according to Duncan's multiple range test. **(B)** Leaves of *Gbvdr6* over-expressed cotton (L1) and wild type inoculated with *V. dahliae in vitro*. The photos were taken at 5 days after inoculation. **(C)** The *Gbvdr6* over-expressed cotton inoculated with *V. dahliae in vivo*. The photos were taken at 15 days after inoculation. **(D)** Assay of disease index of transgenic lines and wild type by the *V. dahliae* isolate BP2. The degree of disease was divided into five grades with disease scores ranging between 0 and 4, and fisher's exact test was conducted to determine whether there is a significant difference between the WT and transgenic lines. The asterisk indicated above the columns means ^**^P < 0.01. **(E)** Fungal biomass upon inoculation with *V. dahliae* isolate BP2. It was determined by qRT-PCR, and the bars represent Verticillium ITS transcript levels relative to the cotton *UBQ14* gene. Data were the means ± SE of three biological replicates and significant differences by Student's test for *P* < 0.01 are indicated by double asterisks. **(F)** Enzyme activity of PAL and CAT of the *Gbvdr6* over-expressed and WT plants after the inoculation of *V. dahliae* isolate BP2. Duncan's multiple range test was conducted, and the different letters in graphs indicate significant differences (*P* < 0.05).

The resistance conferred by *Gbvdr6* overexpression was further investigated in 5 *Gbvdr6* transgenic cotton lines. Three of these lines (L-1/5/9) significantly overexpressed *Gbvdr6* (Figure 7A). The leaves (Figure 7B) and seedlings (Figure 7C) of wild-type plants and plants of the T3 transgenic line L1 were inoculated *in vitro* with the *Verticillium* isolate BP2. Four days after inoculation of the leaves *in vitro*, the leaves of wild-type plants showed severe chlorosis; in contrast, the leaves of L1 displayed only a small amount of yellowing at 5 dpi (Figure 7B). At 30 dpi, the wild-type seedlings displayed chlorosis and necrosis, whereas only a few leaves of the L1 plants displayed a yellow phenotype (Figure 7C). The disease index of the five transgenic lines and the wild-type plants was also evaluated. At 30 dpi, the disease index of the wild-type plants was approximately 56, whereas that of the *Gbvdr6*-overexpressing (OE) plants ranged from 22.1 to 45.2 (Figure 7D). The fungal biomass present in the *V. dahliae*-infected plants was further analyzed by qPCR. The biomass of *Verticillium* was approximately 8-fold higher in the wild-type plants than in the L1 plants at 30 dpi (Figure 7E), confirming that *Gbvdr6* overexpression confers resistance to *Verticillium* in cotton. In addition, the activities of catalase (CAT) and phenylalanine ammonia lyase (PAL) were measured; the activities of the two enzymes in wild-type and L1 transgenic plants showed no difference before infection. The activity of these enzymes was much higher in the transgenic line L1 than in the wild-type strain at 1–7 days after infection (Figure 7F).

**Figure 8:**
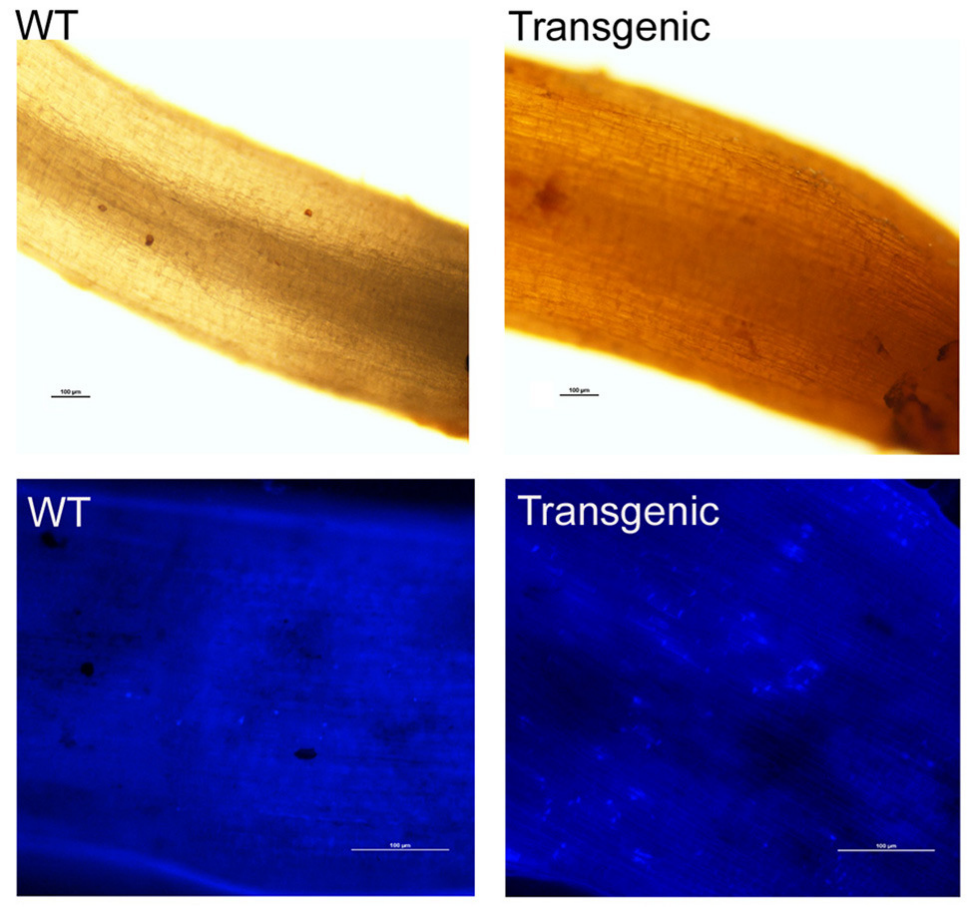
Hydrogen peroxide and callose accumulation in the *Gbvdr6* over-expressed cotton and WT plant in response to *V. dahliae*. Roots from the transgenic and the WT at 5 days post-inoculation with *V. dahliae* were stained with 3,3′-diaminobenzidinetetrahydrochloride (DAB), and photos were taken under a fluorescence microscope with bright light. Scale bar = 100 μm **(upper)**. Callose accumulation **(lower)**. Roots from the transgenic and the WT at 5 days post-inoculation with *V. dahliae* were stained with aniline blue, and photos were taken under a fluorescence microscope with UV light. Scale bar = 100 μm.

The accumulation of reactive oxygen species (ROS) in the host during the early stage of pathogen infection is helpful in fighting against pathogens and in activating cellular programs that help the plant cope with pathogens. At 5 days post-inoculation (dpi) with *V. dahliae*, the roots of *Gbvdr6*-overexpressing cotton accumulated significantly higher levels of H_2_O_2_ than those of WT plants (Figure 8, **upper**). Callose deposition is another immune response to pathogen challenge. At 5 dpi, the roots of *Gbvdr6*-overexpressing cotton deposited more callose at the site of infection than those of wild-type *Arabidopsis* (Figure 8, **lower**).

## Discussion

We used the genome walking method to clone the full-length sequence of *Gbvdr6* from *G. barbadense* Hai7124 cDNA and genomic DNA. Sequence analysis of *Gbvdr6* cDNA and DNA revealed no introns within this gene, similar to the reported Verticillium wilt-resistance genes *Ve1* in tomato and *GbVe, GbVe1*, and *Gbvdr5* in *G. barbadense* (Kawchuk et al., 2001; Zhang et al., 2011; Zhang B. et al., 2012; Yang et al., 2015a). The release of the genome sequence of upland cotton (*G. hirsutum*) cv. TM-1 (Li et al., 2015) made it possible for us to compare the *Gbvdr6* gene from *G. barbadense* Hai7124 with that of a Verticillium wilt-sensitive strain. Twenty-two nucleotides of the *Gbvdr6* gene sequence and seventeen amino acids of the Gbvdr6 protein differed between the resistant strain cv. Hai7124 and the susceptible strain cv.TM-1, suggesting that the three-nucleotide deletion in *Gbvdr6* of Hai7124 did not affect its translation (Figure S1). Single-nucleotide polymorphisms between the resistant and susceptible cultivars have also been detected in cotton *GbVe* and *Gbvdr5* (Zhang et al., 2011; Yang et al., 2015a) and tomato *Ve1* (Fradin et al., 2009). However, the amino acid sequences of *GbVe* in the resistant cv. Pima 90-53 and *GhVe* in the susceptible cv. CRI8 are identical (Zhang et al., 2011). In contrast, compared with *Gbvdr5* from the resistant cv. Hai7124, a single-nucleotide deletion that results in termination of translation was found in *Ghvdr5* from the susceptible cotton genotype Yuman1 (Yang et al., 2015a). The *Ve1* sequence also differs in resistant and susceptible tomato cultivars, with a single nucleotide deletion accompanied by premature termination in susceptible tomato genotypes (Fradin et al., 2009). Multiple alignment analysis further showed that the Gbvdr6 protein shared high homology with other Ve1-homologous RLPs in cotton. These cotton Ve1 homologs possess similar domains, such as a signal peptide domain, multiple eLRRs, a transmembrane domain and a cytoplasmic tail. These characterized domains, particularly the eLRRs, appear to provide a structural framework for functionality in Verticillium wilt resistance (Zhang et al., 2011). Recent studies of the functional determinants of Ve1 through domain swapping and site-directed mutagenesis have revealed that three consecutive eLRR regions (i.e., eLRR1-eLRR8, eLRR20-eLRR23, and eLRR32-eLRR37), the non-LRR island domain (C2), the transmembrane region and the cytoplasmic tail are critical for Ve1 functionality (Fradin et al., 2014; Zhang Z. et al., 2014). However, the Gbvdr6 domains and the regions responsible for Verticillium wilt resistance remain to be further investigated.

*V. dahliae* is a soil-borne pathogen that can successfully penetrate the root epidermis to invade the cortical tissues and cause systemic infection in plants (Klosterman et al., 2009). Analysis of the expression of *Gbvdr6* in various tissues revealed that the highest expression occurs in cotton root, which may allow the plant to set up a protective barrier against infection by *V. dahli*ae. The locations of proteins are critically linked to their functionality. In this study, Gbvdr6 was found to be localized to the plasma membrane, as expected. This is consistent with the results of previous studies in which the proteins encoded by Verticillium wilt-resistance genes such as *Ve1, GbVe*, and *Gbvdr5* were all shown to be localized to the plasma membrane (Zhang et al., 2011; Fradin et al., 2014; Yang et al., 2015a).

The strong homology between *Gbvdr6* and the reported genes encoding cotton RLPs, including *GbVe* (Zhang et al., 2011), *GbVe1* (Zhang B. et al., 2012), and *Gbvdr5* (Yang et al., 2015a), enabled us to investigate their physical locations. Notably, *Gbvdr6, GbVe1* and *Gbvdr5* were found to be physically clustered on the same chromosome. Previous studies have shown that *Cf* genes are highly homologous and are generally located in clusters (Kruijt et al., 2005). A few *cf* homologs have protective functions against *C. fulvum*, whereas others may serve as a reservoir of novel *Cf* genes through sequence exchange between homologs (Kruijt et al., 2005). Dozens of Verticillium wilt-resistance QTLs have been detected in most of the 26 tetraploid cotton chromosomes of various cotton populations (Zhang J. et al., 2014). This enabled us to investigate whether *Gbvdr6* is related to the known Verticillium wilt- resistance QTLs. Interestingly, a Verticillium wilt-resistance QTL hotspot was identified adjacent to a gene cluster with homology to Gbvdr6-Gbvdr5-GbVe1 in the A01 (C1) and D01 (c15) chromosomes of tetraploid cotton, supporting the idea that this gene cluster is vital to Verticillium wilt resistance.

Transcriptome profiling of *G. barbadense* inoculated with *V. dahliae* revealed large accumulations of defense-related transcripts, including transcripts of the *PR1, PR2, PR3, PR5*, and *BAG*-like genes and transcripts encoding dynamin-related proteins (Zhang et al., 2013), suggesting a role in resistance to *V. dahliae*. Overexpression of *Gbvdr6* in *Arabidopsis* also enhanced the expression of the pathogenesis-related genes *PR1, PR2, PR5, PR3, ERF1*, and *PDF1.2*, and these genes were further upregulated when transgenic *Gbvdr6 Arabidopsis* was subjected to *V. dahliae* infection. Because *PR1, PR2* and *PR5* are marker genes for SA signaling and *PR3, ERF1*, and *PDF1.2* are marker genes for JA/ET signaling (Mazarei et al., 2007), the findings suggest that *Gbvdr6* plays a role in activating both SA signaling and JA/ET signaling. In contrast, the expression of the marker gene *GST2* in ET signaling was similar in transgenic and WT plants, even when the plants were infected with. *V. dahliae*. This finding is inconsistent with previous results obtained in *Gbvdr5*- and *GbVe1*-overexpressing transgenic *Arabidopsis*, in which *GST1* was significantly more upregulated in the transgenic than in the WT plants after *V. dahliae* infection (Zhang B. et al., 2012; Yang et al., 2015a).

The relationship between SA and JA is not always antagonistic and may sometimes be collaborative (Mur et al., 2006; Liu et al., 2016). Moreover, enhanced JA levels concomitant with increased SA production and heightened expression of SA-responsive *PR* genes have been found under some conditions (Thaler et al., 2012; Tong et al., 2012). A noncanonical mechanism has been reported in which the JA signaling pathway can be activated following SA accumulation and through the SA receptors NPR3 and NPR4 (Liu et al., 2016). In addition, Verticillium wilt is a kind of hemibiotrophic pathogen that colonizes its hosts as a biotroph during the first part of its life cycle in the xylem, killing them during the subsequent necrotrophic phase (Klosterman et al., 2011). The SA- and JA-dependent signaling pathways are thought to interact synergistically during plants' responses to hemibiotrophic pathogens (Edgar et al., 2006; Pan et al., 2014; Yi et al., 2014; Zhu et al., 2017). The secreted isochorismatase *VdIsc1* contributes to the full pathogenesis of *Verticillium dahliae* by impairing SA synthesis (Liu et al., 2014). Plants infected with wild-type *V. dahliae* isolates accumulate more free JA and SA than those infected with the *icsh1* mutant (Zhu et al., 2017). *VdSCP7* of *V. dahliae* can activate both SA and JA signaling, and such signaling alters the plants' susceptibility to *Botrytis cinerea* and *Phytophthora capsici* (Zhang et al., 2017).

Unlike wild-type plants, the *Gbvdr6*-overexpressing plants in our study were not sensitive to MeJA, suggesting that the JA signaling pathway in these plants may be blocked; however, JA-sensitive genes were more highly expressed in the transgenic plants. These facts seem contradictory and puzzling. The JA signaling pathway is complex, and some mutants, such as *coi1* and *jin1*, both of which are insensitive to MeJA, show discrepancies. However, *PDF1.2* was observed to be induced much more strongly in *jin1-1* plants throughout the infection process, whereas it was repressed in *coi1* plants. In addition, *JIN1* has been shown to negatively regulate the expression of *PDF1.2* in response to MeJA treatment (Boter et al., 2004; Lorenzo et al., 2004; Laurie-Berry et al., 2006). The *Gbvdr6*-overexpressing plants were insensitive to JA but expressed high levels of PDF1.2, suggesting that a mediator such as *JIN1* may be repressed and that this repression may be involved in the resistance mediated by *Gbvdr6*. In addition, the *Arabidopsis* mutant *cev1* has constitutively active JA/ET signal pathways but is insensitive to JA, as shown by the fact that its morphological traits were unchanged when treated with JA (Ellis and Turner, 2001).

In response to pathogens, plants have evolved a series of inducible responses including HR, the production of ROS and callose, and the production and accumulation of PR proteins, phytoalexins, and antimicrobial proteins (Luo et al., 2014). *Ve1*-mediated *Verticillium* resistance was activated by the *V. dahliae* effector protein Ave1 and triggered an HR in tomato (Jonge et al., 2012). However, *Ve1*-mediated HR may be determined by the plant species because no HR was found in *N. benthamiana* or *Arabidopsis*, suggesting that the HR is not absolutely required for Verticillium wilt resistance (Zhang et al., 2013a,b). Moreover, *Ve1*-mediated resistance involved the induction of ROS and increased the activities of peroxidase, phenylalanine ammonia lyase, and lignins (Gayoso et al., 2010). The significant induction of ROS in *Gbvdr6*-overexpressing *Arabidopsis* at the early time points of *Verticillium* infection is consistent with the findings for *Ve1*. The rapid increase in ROS levels observed in transgenic *Gbvdr6 Arabidopsis* plants is likely related to several of the plant's known defense functions, including the production of a compound that is toxic to microbes and the participation of ROS in cell wall reinforcement, lipid peroxidation, signal transduction cascades and the triggering of defensive responses (Gayoso et al., 2010). Callose deposition facilitates the host's defenses against pathogen penetration at early time points of infection (Blümke et al., 2013; Ellinger et al., 2013). The deposition of higher amounts of callose in *Gbvdr6*-overexpressing cotton roots at 5 days post-inoculation suggested increased resistance to *V. dahliae* penetration. This finding is consistent with previously reported findings for *Gbvdr5*, the overexpression of which caused more callose to be deposited at the site of infection (Yang et al., 2015a).

Our observations suggest that *Gbvdr6* contributes to Verticillium wilt resistance. *Gbvdr6* is a receptor-like protein located on the plasma membrane and has been suggested to detect extracellular microbe-derived ligands. Receptor-like proteins are hypothesized to interact with receptor-like kinases (RLKs) or other membrane proteins to activate downstream signaling (Jeong et al., 1999; Humphries et al., 2011; Yang et al., 2012; Postma et al., 2016). However, although the increased survival rate of *Gbvdr6*-overexpressing *Arabidopsis* plantlets after *V. dahliae* infection suggests a role of *Gbvdr6* in defense against pathogens, the transgenic plants were not immune to this pathogen, unlike transgenic *Ve1 Arabidopsis*, which was immune to infection by the *V. dahliae* isolate race1 (Fradin et al., 2011). The overexpression of *Gbvdr6* in *Arabidopsis* activated marker genes for SA signaling and JA/ET signaling and triggered ROS production and callose deposition at early time points after infection in transgenic cotton. *Gbvdr6* was physically clustered with *GbVe1* and *Gbvdr5*, another two Verticillium wilt-resistance genes on the same chromosomes in tetraploid cotton. However, the relationships between these RLP gene clusters and their nearby Verticillium wilt-resistance QTLs on the same chromosomes remain to be further explored.

## Author contributions

YY and XL performed the laboratory experiments and analyzed data, TC performed bioinformatics analyses. All authors contributed to experimental design and conceived experiments. YY and ZM wrote the manuscript and designed figures and schematics.

### Conflict of interest statement

The authors declare that the research was conducted in the absence of any commercial or financial relationships that could be construed as a potential conflict of interest.

## Supplementary material

Figure S1 Alignment of the *Gbvdr6* of Hai7124 and its homology in TM-1. The red letters indicate the variant amino acids between the two genes.
