# Supplementary material for: Gbvdr6, a Gene Encoding a Receptor-Like Protein of Cotton (Gossypium barbadense), Confers Resistance to Verticillium Wilt in Arabidopsis and Upland Cotton
Source: Front Plant Sci. 2018 Jan 17;8:2272. doi: 10.3389/fpls.2017.02272 (PMC5776133; doi:10.3389/fpls.2017.02272)
Supplement: Figure S1 — Alignment of the Gbvdr6 of Hai7124 and its homology in TM-1. The red letters indicate the variant amino acids between the two genes. [file Image1.PDF]

```
Gbvdr6        CTCAACCTAGTGCCATTGTTATCTTCTTCATAACTCTTTGTTGTATTGATATCTCTCATTATTTTGTATTGATATCTCTCATTATTGATACTA
TM-1 homolog  CTCAACCTAGTGCCATTCTTATCTTCTTCATAACTCTTTGTTGTATTGATATCTCTCATTATTTTGTATTGATATCTCTCATTATTGATACTA

Gbvdr6        ATGAGGATTTCACTCTTTTCATTGCTTTTCTTGAATTCTTTTGTATCGGTTATGTTTATTGTCGATGTGGTTTTGGTTTCGACTCAATGTCAAAGTGATCAGAGTCGGTTGTTGCTTCAA
TM-1 homolog  ATGAGGATTTCACTCTTTTCATTGCTTTTCTTGAATTCTTTTGTATCGGTTATGTTTATTGTCGATGTGGTTTTGGTTTCGGCTCAATGTCAAAGTGATCAGAGTCGGTTGTTGCTTCAA
               M  R  I  S  L  F  S  L  L  F  L  N  S  F  V  S  V  M  F  I  V  D  V  V  L  V  S  T  Q  C  Q  S  D  Q  S  R  L  L  L  Q
Gbvdr6        CTCGAAAGCAGCTTCAGCTACAATTATGATTCATCAGGAAAGCTGGTGCCGCTGAAATGGAATCAAAACACAGATTGTTGTTCCTGGGATGGTGTAAGTTGCGATGGAGGTGGTCATGTC
TM-1 homolog  CTCGAAAGCAGCTTCAGCTACAATTATGATTCATCAGGAAAGCTGGTGCCGCTGAAATGGAATCAAAACACAGATTGTTGTTCCTGGGATGGTGTAAGTTGCGATGGAGGTGGTCATGTC
               L  E  S  S  F  S  Y  N  Y  D  S  S  G  K  L  V  P  L  K  W  N  Q  N  T  D  C  C  S  W  D  G  V  S  C  D  G  G  G  H  V
Gbvdr6        ATCGGTCTTGACTTGAACAGGAGATCAATTTCAAGTTCAATTGACAATTCAAGTAGTCTTTTTCGTCTTCAACATCTTCAGTGGCTCAATTTGGCTTATAACGAATTCAAGCCAGCTTTT
TM-1 homolog  ATCGGTCTTGACTTGAACAGGAGATCAATTTCAAGTTCAATTGACAATTCAAGTAGTCTTTTTCGTCTTCAACATCTTCAGTGGCTCAATTTGGCTTATAACGAATTCAAGCCAGCTTTT
               I  G  L  D  L  N  R  R  S  I  S  S  S  I  D  N  S  S  S  L  F  R  L  Q  H  L  Q  W  L  N  L  A  Y  N  E  F  K  P  A  F
Gbvdr6        CCTTCTGCGTTTGATAAGCTGGAGAATTTGAGTTATCTTAACTTGTCCCATGCTGGCTTTGAAGGACAAATTCCAATAGAGATATCACGCTTGACAAGGTTGGTTACTCTTGATTTATCT
TM-1 homolog  CCTTCTGCGTTTGATAAGCTGGAGAATTTGAGTTATCTTAACTTGTCCCATGCTGGCTTTGAAGGACAAATTCCAATAGAGATATCACGCTTGCCAAGGTTGGTTACTCTTGATTTATCT
               P  S  A  F  D  K  L  E  N  L  S  Y  L  N  L  S  H  A  G  F  E  G  Q  I  P  I  E  I  S  R  L  T  R  L  V  T  L  D  L  S
Gbvdr6        GTATCTTTATATCTTGGAAGACCATTGAAACTTGAGAAGCCAAACCTAGAGATGCTTGTTCAAAATCTCACGAAGCTGAGATTTCTCTATCTTGATGATGTAAATATATCAGCTAAGGGG
TM-1 homolog  GTATCTTTATATCTTGGAAGACCATTGAAACTTGAGAAGCCAAACCTAGAGATGCTTGTTCAAAATCTCACGAAGCTGAGATTTCTCTATCTTGATGATGTAAATATATCAGCTAAGGGG
               V  S  L  Y  L  G  R  P  L  K  L  E  K  P  N  L  E  M  L  V  Q  N  L  T  K  L  R  F  L  Y  L  D  D  V  N  I  S  A  K  G
Gbvdr6        AACGAGTGGTGCAAGGCTTTATTGCCACTGACCGAGTTGCAAGTGTTGAACATGTCCGGCTGTTATCTATCGGGACCTATACATTCTTCACTTTCCAAGCTTCGATCTCTCTCAGTAATT
TM-1 homolog  AACGAGTGGTGCAAGGCTTTATTGCCACTGACCGAGTTGCAAGTGTTGAACATGTCCGGCTGTTATCTATCGGGACCTATACATTCTTCACTTTCCAAGCTTCGATCTCTCTCAGTAATT
               N  E  W  C  K  A  L  L  P  L  T  E  L  Q  V  L  N  M  S  G  C  Y  L  S  G  P  I  H  S  S  L  S  K  L  R  S  L  S  V  I
Gbvdr6        CACTTGGACTTCAACAACTTGTCTGCGACGGTTCCAAAATTCTTTGCGGAATTCCCAAACCTGACTTCCCTCAGTCTTACTTACACTAATTTGAGTGGAAGATTGCCAGATGAAAATTTT
TM-1 homolog  CACTTGGACTTCAACAACTTGTCTGCGTCGGTTCCAAAATTCTTTGCGGAATTCCCAAACCTGACTTCCCTCAGTCTTACTTACACTAATTTGAGTGGAAGATTGCCAGATGAAATTTTT
               H  L  D  F  N  N  L  S  A  T  V  P  K  F  F  A  E  F  P  N  L  T  S  L  S  L  T  Y  T  N  L  S  G  R  L  P  D  E  N  F
Gbvdr6        CAGATACCTACATTACAGACTCTTGATTTGTCAGACAACGATTTACTCCAAGGTTCGTTTCAAAAATTTTCTCCCAATCTTTCTCTTCAAATTCTGTTACTTAGCCGCACAAATTTTGAG
TM-1 homolog  CAGATACCTACATTACAGACTCTTGATTTGTCAGACAACGATTTACTCCAAGGTTCGTTTCAAAAATTTTCTCCCAATCTTTCTCTTCAAATTCTGTCACTTAGCCGCACAAATTTTGAG
               Q  I  P  T  L  Q  T  L  D  L  S  D  N  D  L  L  Q  G  S  F  Q  K  F  S  P  N  L  S  L  Q  I  L  L  L  S  R  T  N  F  E
Gbvdr6        GGGCAAGTACCAGAATCTCTCGGTAACCTTGGAAAACTGACAAGAATAGAGCTTGCAGAATGTAATTTCAGTGGAGCCATACCCAAAACAATGAAGAAACTTACCCAACTTGTGTATCTG
TM-1 homolog  GGGCAAGTACCAGAATCTCTCGGTAACCTTGGAAAACTGACAAGAATAGAGCTTGCAGAATGTAATTTCAGTGGAGCCATATCCAAAACAATGAAGAAACTTACCCAACTTGTGTATCTG
               G  Q  V  P  E  S  L  G  N  L  G  K  L  T  R  I  E  L  A  E  C  N  F  S  G  A  I  P  K  T  M  K  K  L  T  Q  L  V  Y  L
Gbvdr6        GATTTTTCCTTTAACCGTTTTTCTGGTCCAATACCATCATTCTCATCATCCAGAAATCTTACATATCTAAGTTTTGGTTATAATCAGTTAAATGGTGGAATTCATTCCACTGATTGGTCA
TM-1 homolog  GATTTTTCCTTTAACCGTTTTTCTGGTCCAATACCATCATTCTCATCATCCAGAAATCTTACATATCTAAGTTTTGGTTATAATCAGTTAAATGGTGGAATTCATTCCACTGATTGGTCA
               D  F  S  F  N  R  F  S  G  P  I  P  S  F  S  S  S  R  N  L  T  Y  L  S  F  G  Y  N  Q  L  N  G  G  I  H  S  T  D  W  S
Gbvdr6        AGTCTTTCTGAGCTAGAAATTGCTTACTTAGGAAAGAACAAGTTAAGTGGAACCATTCCACCGGCTTTGTTTTGCGTTCCATCACTGCAAAGACTTTTCCTTTCTCAAAACCAATTCAAA
TM-1 homolog  AGTCTTTCTGAGCTAGAAATTGTTGACTTAGGAAAGAACAAGTTAAGTGGAACCATTCCACCGGCTTTGTTTTGCGTTCCATCACTGCAAAGACTTTTCCTTTCTCAAAACCAATTCAAA
               S  L  S  E  L  E  I  A  Y  L  G  K  N  K  L  S  G  T  I  P  P  A  L  F  C  V  P  S  L  Q  R  L  F  L  S  Q  N  Q  F  K
Gbvdr6        GGTAACCTTAGTGACCTTCATGGTAAGGCCTCTTCATTGCTTGAGGGACTTGATCTTAGTAGCAACAAGTTTCAAGGGCAATTCCCAATGTCCGTGTTTGAACTCCATGGTCTGAAGTTA
TM-1 homolog  GGTAACCTTAGTGACCTTCATGGTAAGGCCTCTTCATTGCTTGAGGGACTTGATCTTAGTAGCAACAAGTTTCAAGGGCAATTCCCAATGTCCGTGTTTGAACTCCATGGTCTGAAGTTA
               G  N  L  S  D  L  H  G  K  A  S  S  L  L  E  G  L  D  L  S  S  N  K  F  Q  G  Q  F  P  M  S  V  F  E  L  H  G  L  K  L
Gbvdr6        CTATCCCTTTCCTCAAACAACTACAGTGGATCGATACCAATGAGTGCCTTTCAGAACTTGAGGAATCTTTCTTACCTTGATCTCTCATATAACAGGTTGTCTATTGATGTCACCGATACT
TM-1 homolog  CTATCCCTTTCCTCGAACAACTACAGTGGATCGATACCAATGAGTGCCTTTCAGAACTTGAGGAATCTTTCTTACCTTGATCTCTCATATAACAGGTTGTCTATTGATGTCACCGATACT
               L  S  L  S  S  N  N  Y  S  G  S  I  P  M  S  A  F  Q  N  L  R  N  L  S  Y  L  D  L  S  Y  N  R  L  S  I  D  V  T  D  T
Gbvdr6        AATATTTCCTCGATTTCTTTCCCCACATTTACCACATTGAAGTTGGCGTCTTGCAACTTAACCGAGTTCCCTGATTTTTTGAAGAATCAGTCCAGTTTAATTCAACTAGACCTCTCAAAG
TM-1 homolog  AATATTTCCTCGATTTCTTTCCCCACATTTACCACATTGAAGTTGGAGTCTTGCAACTTAACCGAGTTCCCTGATTTTTTGAAGAATCAGTCCAGTTTAATTCAACTAGACCTCTCAAAG
               N  I  S  S  I  S  F  P  T  F  T  T  L  K  L  A  S  C  N  L  T  E  F  P  D  F  L  K  N  Q  S  S  L  I  Q  L  D  L  S  K
Gbvdr6        AATCAAATTCATGGGAAAATACCCAATTGGATTTGGAAAGCAACAAGTCTCGAGTACCTAAATCTTTCTCAGAACTTTTTTGTGGAATTTCAAAGACCTTTGGAGAATATAACTTCTAAT
TM-1 homolog  AATCAAATTCATGGGAAAATACCCAATTGGATTTGGAAAGCAACAAGTCTCGAGTACCTAAATCTTTCTCAGAACTTTTTTGTGGAATTTCAAAGACCTTTGGAGAATATAACTTCTAAT
               N  Q  I  H  G  K  I  P  N  W  I  W  K  A  T  S  L  E  Y  L  N  L  S  Q  N  F  F  V  E  F  Q  R  P  L  E  N  I  T  S  N
Gbvdr6        GTTCGTTTTCTTGACGCACATGGGAGCCAATTGCAAGGGCAAATCCCAATTCTTAACCCATATGATGTCTTTTATCTGGATTACTCTTCTAACAATTTCAGCTCCATTTTACCACCTCGC
TM-1 homolog  GTTCGTTTTCTTGACGCACATGGGAACCAATTGCAAGGGCAAATCCCAATTCTTAACCCATATGATGTCTTTTATCTGGATTACTCTTCTAACAATTTCAGCTCCATTTTACCACCTCAC
               V  R  F  L  D  A  H  G  S  Q  L  Q  G  Q  I  P  I  L  N  P  Y  D  V  F  Y  L  D  Y  S  S  N  N  F  S  S  I  L  P  P  R
Gbvdr6        ATTGGTGACTCCCTCCGGTCTGCTTCTTTCTTGTCCCTTTCAAATAATAACTTTCATGGGAGTATCCCTCAGTCGATATGCAATAGTACATCTCTTGATGTACTTGATCTGTCTAATAAT
```

```
TM-1 homolog   ATTGGTGACTCCCTCCGGTCTGCTTCTTTCTTGTCCCTTTCAAATAATAACTTTCATGGGAGTATCCCTCAGTCGATATGCAATAGTACATCTCTTGATGTACTTGATCTGTCTAATAAT
                I  G  D  S  L  R  S  A  S  F  L  S  L  S  N  N  N  F  H  G  S  I  P  Q  S  I  C  N  S  T  S  L  D  V  L  D  L  S  N  N
Gbvdr6         TCCCTGAGTGGCCCAATTCCTCAATGCCTCTTTCAGATGAACGTGTCTCTTGGAGTACTGAATCTAGGAGGAAACAATCTCAGTGGCATCATTTCTGACACTTTTCCAGAAAGTTGTAAG
TM-1 homolog   TCCCTGAGTGGCCCAATTCCTCAATGCCTCTTTCAGATGAACGTGTCTCTTGGAGTACTGAATCTAGGAGGAAACAATCTCAGTGGCATCATTTCTGACACTTTTCCAGAAAGTTGTAAG
                S  L  S  G  P  I  P  Q  C  L  F  Q  M  N  V  S  L  G  V  L  N  L  G  G  N  N  L  S  G  I  I  S  D  T  F  P  E  S  C  K
Gbvdr6         TTGCAAACTCTAGATCTTAATCAGAACCGATTGGGAGGAAAGGTTCCAAAATCATTGGGGAATTGCAAAATGCTGGAGGTTTTAGACATTGGCACCAATCAGATCAACGGCAATTTCCCA
TM-1 homolog   TTGCAAACTCTAGATCTTAATCAGAACCGATTGGGAGGAAAGGTTCCAAAATCATTGGGGAATTGCAAAATGCTGGAGGTTTTAGACATTGGCACCAATCAGATCAACGGCAATTTCCCA
                L  Q  T  L  D  L  N  Q  N  R  L  G  G  K  V  P  K  S  L  G  N  C  K  M  L  E  V  L  D  I  G  T  N  Q  I  N  G  N  F  P
Gbvdr6         TGCCATTTGAAGAATATAGCCACGCTGCATGTCCTTATTTTACGATCCAACAAATTCAACGGTCACATTGATTGTCCGGGAAACAATAGTGGATGGCCATTGCTTCAGATTTTTGACTTA
TM-1 homolog   TGCCATTTGAAGAATATAGCCACGCTGCATGTCCTTATTTTACGATCCAACAAATTCAACGGTCACATTGATTGTCCGGGAAACAATAGTGGATGGCCATTGCTTCAGATTTTTGACTTA
                C  H  L  K  N  I  A  T  L  H  V  L  I  L  R  S  N  K  F  N  G  H  I  D  C  P  G  N  N  S  G  W  P  L  L  Q  I  F  D  L
Gbvdr6         GCATCCAACAATTTTAGTGGTAAATTGCATCTATCTGGTTTGGGGACCTGGGAGGCTATGCGGTCTAATCAAGATAAAAACCAATCAAAGCTCAAACATCTCATGTTTGACCTCTTAGAA
TM-1 homolog   GCATCCAACAATTTTAGTGGTAAACTGCATCTATCTGGTTTGGGGACCTGGGAGGCTATGCGGTCTAATCAAGATAAAAACCAATCAAAGCTCAAACATCTCATGTTTGACCTCTTAGAA
                A  S  N  N  F  S  G  K  L  H  L  S  G  L  G  T  W  E  A  M  R  S  N  Q  D  K  N  Q  S  K  L  K  H  L  M  F  D  L  L  E
Gbvdr6         GATGATGACCAATACTACTATCAAGATGAAATAACAGTTACCATCAAAGGCAATGAGTTGGAGCTGGTGAAAATCTTGACCGTGTTCACTTCCATTGACATTTCATGCAACAACTTTGAA
TM-1 homolog   GATGATGACCAATACTACTATCAAGATGAAATAACAGTTACCATCAAAGGCAATGAGTTGGAGCTGGTGAAAATCTTGACCGTGTTCACTTCCATTGACATTTCATGCAACAACTTTGAA
                D  D  D  Q  Y  Y  Y  Q  D  E  I  T  V  T  I  K  G  N  E  L  E  L  V  K  I  L  T  V  F  T  S  I  D  I  S  C  N  N  F  E
Gbvdr6         GGGCCAATACCAGAAGTAATCGGCAGATTCAACGCACTTTATGCCCTCAACTTTTCACATAATGCTTTCACAGGTTCAATCCCATCATTTT---GGAAAATGCAACACCTTGAGTCCTTG
TM-1 homolog   GGGCCAATACCAGAAGTAATCGGCAAATTCAACGCACTTTATGCCCTCAACTTTTCAGATAATGCTTTCACAGGTTCAATCCCATCATTTTTTGGAAAATTGCAACACCTTGAGTCCTTG
                G  P  I  P  E  V  I  G  R  F  N  A  L  Y  A  L  N  F  S  H  N  A  F  T  G  S  I  P  S  F  W  -  K  M  Q  H  L  E  S  L
Gbvdr6         GATCTCTCAAGCAACAGCCTACGTGGTGAGATCCCTTTGCAGCTAGCAAACCTCAATTTCCTTTCATTTCTCAACGTCTCAAATAATAAGCTAGTGGGTCCAATCCCAACGAGCACCCAA
TM-1 homolog   GATCTCTCAAGCAACAGCCTACGTGGTGAGATCCCTTTGCAGCTAGCAAACCTCAATTTCCTTTCATTTCTCAACGTCTCAAATAATAAGCTAGTGGGTCCAATCCCAACGAGCACCCAA
                D  L  S  S  N  S  L  R  G  E  I  P  L  Q  L  A  N  L  N  F  L  S  F  L  N  V  S  N  N  K  L  V  G  P  I  P  T  S  T  Q
Gbvdr6         CTTCAATCATTTTCAGAAGCTTCATTTGAGAACAATACCGGATTATGTGGACCTCCTTTGAAGACAAAGTGCGGATCGTCACCACCTAAAGAAGACAGCCCTTCAGATTCTGAGACAGGG
TM-1 homolog   CTTCAATCATTTTCAGAAGCTTCATTTGAGAACAATACCGGATTATGTGGACCTCCTTTGAAGACAAAGTGCGGATCCTCACCACCTAAAGAAGACAGCCCTTCAGATTCTGAGACAGGG
                L  Q  S  F  S  E  A  S  F  E  N  N  T  G  L  C  G  P  P  L  K  T  K  C  G  S  S  P  P  K  E  D  S  P  S  D  S  E  T  G
Gbvdr6         AGCATTATACAGTGGAATCATTTAAGTGCCGAGATAGGGTTTATCTTTGGGTTGGGAATTATCATTGTACCTCTTATCTATTGGAAGAGATGGAGGATATGGTATTTTGAGCGTGTCGAT
TM-1 homolog   AGCATTATACAGTGGAATTATTTAAGTGCCGAGATAGGGTTTATCTTTGGGTTGGGAATTATCATTGTACCTCTTATCTATTGGAAGAGATGGAGGATATGGTATTTTGAGCGTGTCGAT
                S  I  I  Q  W  N  H  L  S  A  E  I  G  F  I  F  G  L  G  I  I  I  V  P  L  I  Y  W  K  R  W  R  I  W  Y  F  E  R  V  D
Gbvdr6         CGTGCTCTCTCCAGGCTTTTCCCTCGTCTTGGTCGTGAAACCAAAAAGCATGGGAGGAAAGCAAAGCAGAACCAAAGGAGGACCTAG
TM-1 homolog   CGTGCTCTCTCCAGGCTTTTCCCTCGTCTTGGTCGTGAAACCAAAAAGCATGGGAGGAAAGCAAAGCAGAACCAAAGGAGGACCTAG
                R  A  L  S  R  L  F  P  R  L  G  R  E  T  K  K  H  G  R  K  A  K  Q  N  Q  R  R  T
Gbvdr6         CAACTATTGGGAT
TM-1 homolog   CAACTATTGGGAT
```
